# Cellular Landscaping of COVID-19 and Gynaecological Cancers: An Infrequent Correlation

**DOI:** 10.1155/2022/5231022

**Published:** 2022-10-17

**Authors:** Rahul Bhattacharjee, Debanjan Das, Radheka Bhadhuri, Srija Chakraborty, Tanima Dey, Rupam Buragohain, Asim Nath, Kartik Muduli, Pranjan Barman, Rohit Gundamaraju

**Affiliations:** ^1^KIIT School of Biotechnology, Kalinga Institute of Industrial Technology (KIIT-DU), Bhubaneswar, Odisha, India; ^2^St. Xavier's College (Autonomous), Kolkata, West Bengal, India; ^3^Department of Biotechnology, Gauhati UNiversity, Gopinath Bordoloi Nagar, Guwahati 781014, Assam, India; ^4^ER Stress and Mucosal Immunology Lab, School of Health Sciences, University of Tasmania, Launceston, Tasmania, Australia; ^5^Division of Gastroenterology, School of Medicine, Washington University at St Louis, St Louis, MO, USA

## Abstract

COVID-19 resulted in a mortality rate of 3–6% caused by SARS-CoV-2 and its variant leading to unprecedented consequences of acute respiratory distress septic shock and multiorgan failure. In such a situation, evaluation, diagnosis, treatment, and care for cancer patients are difficult tasks faced by medical staff. Moreover, patients with gynaecological cancer appear to be more prone to severe infection and mortality from COVID-19 due to immunosuppression by chemotherapy and coexisting medical disorders. To deal with such a circumtances oncologists have been obliged to reconsider the entire diagnostic, treatment, and management approach. This review will provide and discuss the molecular link with gynaecological cancer under COVID-19 infection, providing a novel bilateral relationship between the two infections. Moreover, the authors have provided insights to discuss the pathobiology of COVID-19 in gynaecological cancer and their risks associated with such comorbidity. Furthermore, we have depicted the overall impact of host immunity along with guidelines for the treatment of patients with gynaecological cancer under COVID-19 infection. We have also discussed the feasible scope for the management of COVID-19 and gynaecological cancer.

## 1. Introduction

**Figure 1:**
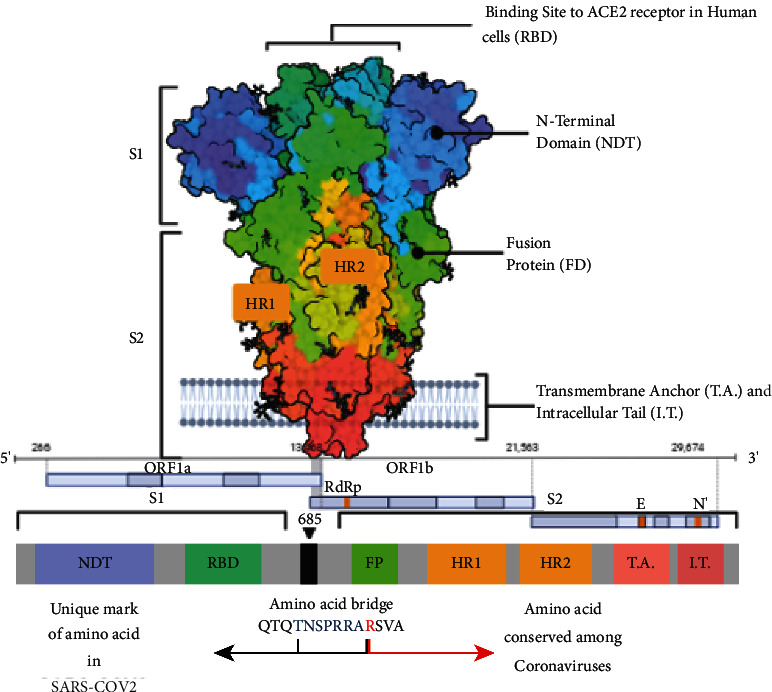
Schematic representation of the domain arrangement of a single spike protein of the SARS-CoV-2 S protein precursor. SS, signal peptide; NTD: N-terminal domain; RBD: receptor-binding domain; RBM: receptor-binding motif; SD1/2: subdomain 1 and 2; FP, fusion peptide; HR1, heptad repeat 1; CH, central helix; CD, connector domain; HR2, heptad repeat 2; TM, transmembrane domain; CT, cytoplasmic tail. The glycans were omitted for clarity. Below it is the 1D structure of the coronavirus spike. NTD, N-terminal domain. FP (fusion peptide), HR1 (heptad repeat 1), and HR2 (heptad repeat 2) are structural units in coronavirus S2 that function in membrane fusion. The sequence of the spike proteins from SARS-CoV-2 is mentioned based on genomic presentation.

**Figure 2:**
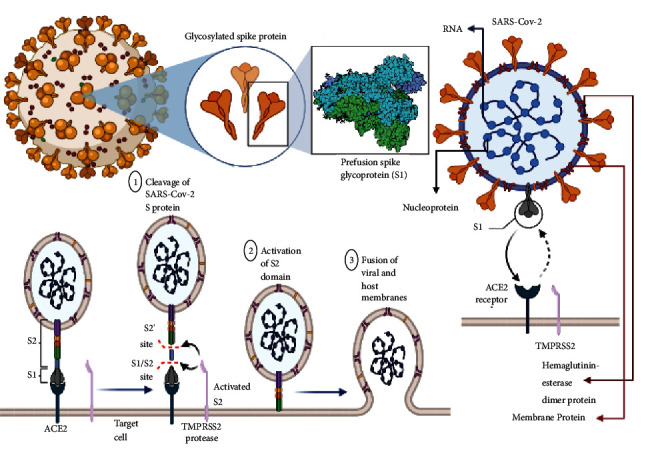
Detailed illustration of structure and mechanism of auxiliary protein, namely, spike proteins inside the genome of SARS-CoV-2 for host-virus interaction. The domains of these glycosylated spike proteins, namely, S1 and S2 are elucidated here. The other membrane and surface proteins include D-dimer protein(D-dimer is one of the protein fragments produced when a blood clot gets dissolved in the body. It is normally undetectable or detectable at a very low level unless the body is forming and breaking down blood clots), haemagglutinin esterase (these are a family of viral envelope glycoproteins that mediate reversible attachment to O-acetylated sialic acids by acting both as lectins and as receptor-destroying enzymes (RDEs)) are also explained here. There is binding of ACE-2 receptor with type 2 pneumocytes for host-virus interaction on the membrane. The primary step involved coupling with the receptor in turn leads to (1) cleavage of SARS-CoV-2 Spike (S) protein, (2) thus causing activation of the S2 domain, and (3) finally leading to the fusion of viral and host membranes.

SARS-CoV-2 virus is an ssRNA (positive sense) virus infection belonging to the family *Coronaviridae* with subfamily (*α*-coronavirus, *β*-coronavirus, *γ*-coronavirus, and *δ*-Coronavirus) which originated for the first time in December 2019 at Wuhan, China. It belongs to the genus *β*-coronavirus, just like the MERS-CoV, and based on the statistical survey until September 8, 2020, there were approximately 27.2 million confirmed cases, along with 890,000 deaths, which had been reported in 187 countries and territories [1]. Fever, myalgia, and exhaustion are the usual COVID-19 symptoms along with headaches, haemoptysis, and septum formation which were thrown in for good measure [2]. Based on the severity of the condition of the patient, it can be divided into critical (5%), infected (81%), and mildly infected with symptoms [3]. Coronaviruses are crown-shaped particles, which have spike proteins on the surface encasing them. The genome of the virus comprises 14 ORFs which encodes 27 proteins with a genomic size of 26–32 kbps explained in Figure 1 [4–6]. The main site of variation would include the spike protein gene and auxiliary gene [7]. The spike protein from the spike gene aids in the host-virus interaction with its two domains, namely, S1 and S2. The S1 domain aids in binding to the receptors on the host cell, whereas the S2 domain with membrane fusion [8]. There is eight auxiliary proteins among which four are structural protein which is found in the 3′ terminus of the genome. This auxiliary protein would also include spike protein, nucleocapsid protein along with matrix protein and envelope protein [6] (Figure 2). An RNA virus is known for its rapid evolution owing to the mechanism of antigenic shift and antigenic drift. The ability of the virus to evolve and virulence modulation are two critical factors for viral adaptability and are linked to a high rate of mutation [9]. It has been demonstrated that the genomes of RNA viruses gain genetic variations while being spread in a single outbreak [10]. Deletion of viral genomic sequences is a natural process that is usually invariably linked to virus attenuation. However, it has the potential to produce a more serious illness [11–13]. Phylogenomic examination of three SARS-CoV-2 strains isolated from epidemics in China, the United States, and Europe revealed no indication of adaptations that are local or regional, within the virus, whereas that viral proteins particularly the S protein may adopt the nucleotide, amino acid, and structural levels [14]. The viral evolution in correlation with the host was connected to the virulence, infectivity, and evolution of the virus due to the immunocompromised status patient [15].

The elderly or the ones with co-morbidities like anorexia, pharyngeal discomfort, diabetes, or hypertension may require ICU care as they are immunocompromised and are highly susceptible in COVID-19 pandemic [16, 17]. Active cancer should be considered as one of the factors that can increase vulnerability as it renders a patient immunocompromised. Since, cancer patients possess a significantly impaired and changed immune systems as a result of various anti-cancer therapy; thus their immunocompromised condition worsens for viral infection. The location of the origin of gynaecological cancer and the severity of the condition has put them in a vulnerable position [18]. Along with the spreading pandemic, gynaecological cancer patient's incidence rates have increased, indicating a bigger number of serious disease cases. COVID-19 patients suffering from cancer had a greater risk than COVID-19 patients without gynaecological cancer [19]. Cancer patients accounted for 5.6% of case fatalities with co-infection of COVID-19 patients [6]. Immunocompromised gynaecological cancer patients have a harder time avoiding respiratory virus infections, rendering them more susceptible to COVID-19 such as viral pneumonia which has been linked to up to 19% of mortality in such patients [6]. Coronavirus pneumonia caused 24% mortality in gynaecological cancer patients with commonly protracted viral shedding, compared to 3% in non-cancer patients [8]. Traditional coronaviruses, in particular, have been linked to increased oxygen requirements and mortality in individuals with haematologic malignancies [20]. Patients suffering from COVID-19 in hospitals have been observed to have lymphopenia, with non-survivors acquiring progressively severe lymphopenia at a greater rate [4], which can lead to pneumonia in patients suffering from haematologic malignancies who also have respiratory virus infections [21, 22].

Here in this review, we have discussed the case reports of gynaecological cancer with COVID-19, pathobiology of COVID-19 in gynaecological cancer, risk, and its effect on host immunity. We have also discussed the molecular link between COVID-19 and gynaecological cancer and its relation with the ACE-2 receptor. Alongside we have discussed the impact of gynaecological cancer on COVID-19 in surgery, diagnosis, and treatment.

## 2. Influence on Host Immunity of Coinfection of COVID-19 along with Cancer Patients

**Figure 3:**
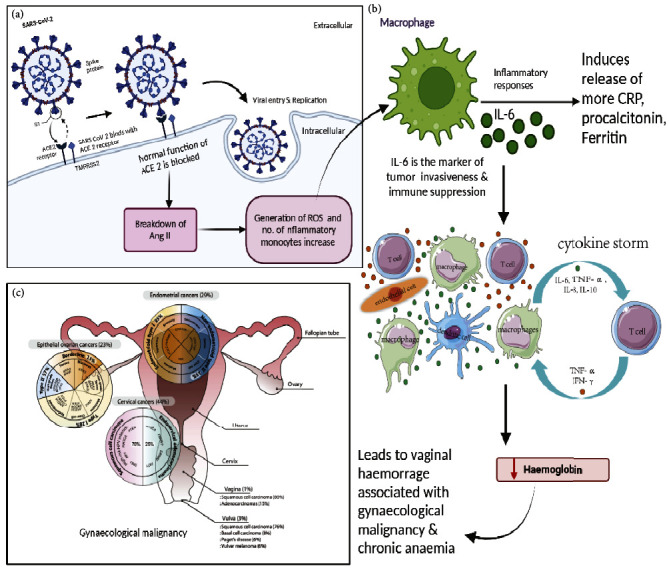
The schematic representation showing the influence of host immunity due to SARS-CoV-2 infection on the gynaecological malignancy. (a) The entry of viral pathogen inside the cell by the interaction of spike protein on the virus and ACE-2 receptor on the cell. The infection influences the breakdown of Ang II since the normal function of the ACE-2 receptor is blocked leading to the generation of ROS and enhanced inflammatory response. (b) IL-6 induces the release of more CRP, procalcitonin, and ferritin which facilitates the coinfection of COVID-19 and malignancy. IL-6 is the marker of immune suppression and influences cytokine storm which in turn reduces the haemoglobin in the blood. (c) Decreased haemoglobin leads to vaginal haemorrhage associated with gynaecological malignancy and indicates chronic anaemia.

Patients suffering from cancer are considered a high-risk group for SARS-CoV-2 infection, and they may experience a variety of COVID-19 symptoms as illustrated in Figure 3 [23]. There is an expectation of a third wave of infections due to the worldwide confirmed cases of COVID-19 with more than 32 million occurrences. The connections between COVID-19 infection and oncologic illness must be understood. There is a significant death rate among patients with co-infection of COVID-19 and gynaecological cancer [24, 25]. Current research is primarily focused on SARS-inflammatory -2 which has consequences that stem from acute lung damage and respiratory distress along with ARDS caused by SARS-CoV-2. Desquamated epithelial cells, some of which are big and contain syncytial nuclei, are seen in the alveolar cavities of SARS-CoV-2 patients [11]. The cytopathogenic impact of viral replication in cells is mediated via the syncytial nuclei [12]. SARS-CoV-2 infection is thought to reduce ACE-2 receptor activation because viruses competitively bind to these receptors, rendering them less useful. The breakdown of Ang II protein, which leads to the production of the vasodilator angiotensin, is one of the most significant activities of ACE-2 receptors [13]. Ang II causes inflammation by triggering the productionof reactive oxygen species (ROS), C-reactive protein, and inflammatory monocytes [26]. The use of ACE-2 during SARS-CoV-2 infection causes a strong inflammatory response in the body, resulting in severe inflammation and potentially fatal symptoms. When the virus causes a multi-system inflammatory syndrome, it has led to serious and life-threatening diseases in both children and adults [27]. Inflammatory indicators such as CRP, procalcitonin, ESR, interleukin-6 (IL-6), and ferritin are seen in COVID-19 patients. Higher IL-6 levels have also been linked to increased COVID-19 mortality [28]. IL-6 receptors, also known as a hallmark of invasiveness and immune suppression, lead to recurrent inflammation accompanied with enhanced chemotherapeutic resistance, metastasis, and epithelial to mesenchymal transition (EMT) [29]. The overreaction of the immune system to COVID-19 causes a cytokine storm, which leads to rapid clinical deterioration, ARDS, and multiorgan failure [30]. Ongoing studies are looking into the function of anti-inflammatory drugs like corticosteroids (NCT04344288) and immune modifying drugs like nivolumab (NCT04343144) in COVID-19 therapy in cancer patients.

Because cancer is known to affect inflammatory and immunologic processes in the body, it is unclear which concepts may be applied to oncology patients with COVID-19 infection. Systemic inflammation has been seen in several forms of gynaecological cancer and has been linked to worse survival results [31, 32]. Chronic pancreatitis, chronic inflammation, inflammatory bowel disease, and hepatitis are all associated with elevated cancer risk [32, 33]. Conversely, during tumour growth, invasion, and metastasis, tumour cells and the TME might express cytokines and other innate immune signaling molecules [32]. Due to the compensatory activation of immune suppressor cells, chronic inflammation might result in an immune-suppressed environment [34]. Patients with a weak tumour-directed immune response have a poorer prognosis than those with a strong immune response [35]. Anticancer therapies can potentially cause systemic immunodeficiency in oncology patients [36]. Certain inflammatory markers which include ferritin, procalcitonin, and C-reactive protein were significantly higher when patients with coinfection of COVID-19 and gynaecological cancer were admitted to the hospital. A significant link between haematologic indicators and COVID-19 severity and mortality was identified in a WBC and D-dimer-dependent manner. When inflammatory markers from patients with active gynaecological cancer and those in remission were compared, no significant difference in the levels were found. However, there was a decrease in the level of haemoglobin in coinfected patients. A substantial reduction in haemoglobin in patients with active malignancies can be ascribed to several factors, including vaginal haemorrhage associated with gynaecologic malignancy, chronic illness anaemia, or chemotherapy-induced anaemia [37–39].

## 3. Risks of Gynaecological Cancer and COVID-19

**Figure 4:**
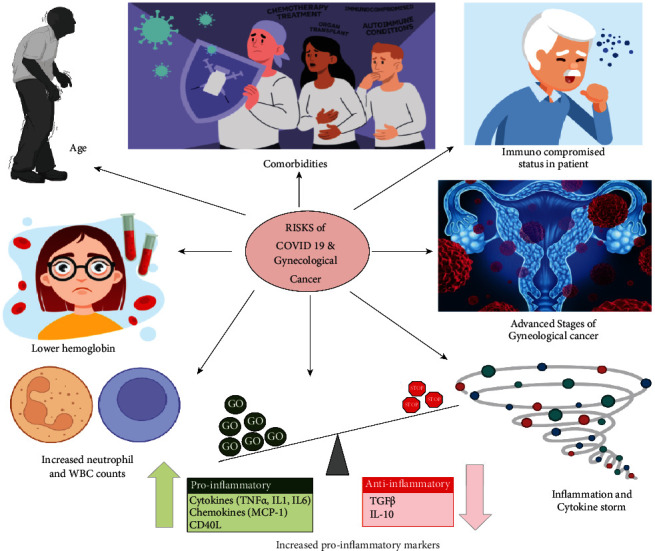
The illustration indicating all the probable risks associated with COVID-19 and gynaecological cancer.

According to the reports of the National Cancer Institute, coronaviruses may have primarily been involved in the development of various malignancies [40]. When it comes to viruses that impact the respiratory tract, gynaecological patients suffering from cancer are expected to grow more complications as compared to noncancer patients, which is consistent with the observation in the case of MERS-CoV infection, that shows a significant rise in the death rate as illustrated in Figure 4 [41]. COVID-19 is approximately 76% more severe in patients suffering from cancer compared to individuals not suffering from cancer [42].  Table 1 illustrates the comparison of clinical features distinguishing noncancer patients with gynaecological cancer patients.

It has been established that the interlink between the risk of COVID-19 infection and the complexity of gynaecological cancer in patients is a result of the patient's immunocompromised status [50]. Patients undergoing cancer treatment are frequently given either immune stimulating or immunosuppressive drugs, depending on the patient and the therapy. In both situations, it causes immunological suppression, making the patient immunocompromised [51]. The severe conditions found in cancer patients as a consequence of infection with SARS-CoV-2 are produced by an increase in innate immune cells, particularly cytokines that promote infiltration of lymphocytes and neutrophils as well as inflammation. This phenomenon is termed a “cytokine storm.” As a result of the cytokine storm, there has been an increase in the incidence of fatalities in patients who had both COVID-19 infection and malignancy. Inflammation affects patients with gynaecologic cancer and COVID-19 and since gynaecologic cancer patients were suffering from systemic inflammation, the condition deteriorates [52].

The age of the patient can also be a significant risk factor in gynaecological cancers, making the patients more vulnerable to COVID-19 infection. The capacity of a gynaecological cancer patients to establish natural immunity to COVID-19 is influenced by age. The stage of the disease may also be a substantial risk factor in cancer patients, and some patients may be undergoing radiation or have undergone surgical operations [52, 53]. COVID-19-related mortality did not differ substantially between individuals undergoing chemotherapy or radiation treatment and those who did not. Co-morbidities in cancer patients suffering from diseases such as heart diseases, congestive heart failure, hypertension, and lung diseases can increase mortality caused due to COVID-19 [53, 54]. Moreover, patients who were deceased with coinfection of gynaecological cancer and COVID-19 exhibited lower haemoglobin, increased neutrophil and WBC counts, and increased proinflammatory markers such as lactate dehydrogenase and D-Dimer as compared to those who recovered from the coinfection [55]. In addition, comparing SARS-CoV-2 cases in general as well as sex and age-matched, it was seen that noncancer patients suffering from SARS-CoV-2, have a lower mortality rate as compared to patients suffering from coinfected patients with gynaecological cancer and COVID-19, in all age groups. This indicates that the effect of COVID-19 on patients suffering from gynaecological cancer is much more severe compared to a healthy population [56, 57].

## 4. Molecular Link between COVID-19 and Gynaecological Cancer

The virus enters the human body through the respiratory tract and reaches the lungs and upon entry inside the host system via air droplet, it binds in an ACE-2 and TMPRSS2-dependent manner to the type 2 pneumocytes [58]. The presence of spike proteins on the virus aids in the binding of ACE-2. The binding of the virus to the type 2 pneumocytes occurs in a spikes protein-dependent manner causing its multiplication inside the host system. Upon increased viral load inside the host system, the virus disseminates in an exocytosis-dependent manner leaving the cells and the bloodstream and causing the release of proinflammatory cytokines which would include IL-7, IL-8, IL-9, IL-10, TNF-alpha, and IFN-gamma via the immune cells of the host body. These infection characteristics can be linked to cell pyroptosis and chemokines, as well as increasing macrophage and alveolar secretion and initiating pyroptosis [59].

**Figure 5:**
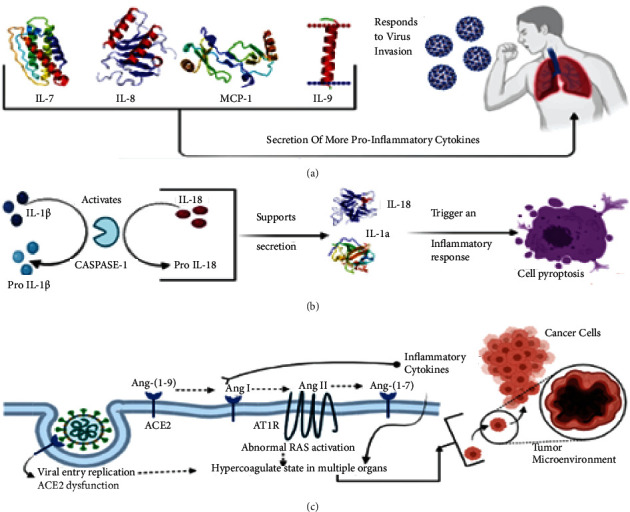
Schematic overview of a molecular link between COVID-19 coinfection with gynaecological cancer in patients. (A) The cascade of molecular macromolecules that is triggered by the host immune response upon entry of SARS-CoV-2 and responds to virus invasion by secreting proinflammatory cytokines and metalloprotease which include IL-7, IL-8, IL-9, and MCP-1. (B) This cascade of events causes caspase-1 to activate IL-1beta and IL-18 and upon activation it triggers an inflammatory response leading to cell pyroptosis. (C) Illustration of how coagulation in multiple organs reaches tumour microenvironment due to ACE-2 dysfunction and abnormal RAS activation by causing hypercoagulation.

**Figure 6:**
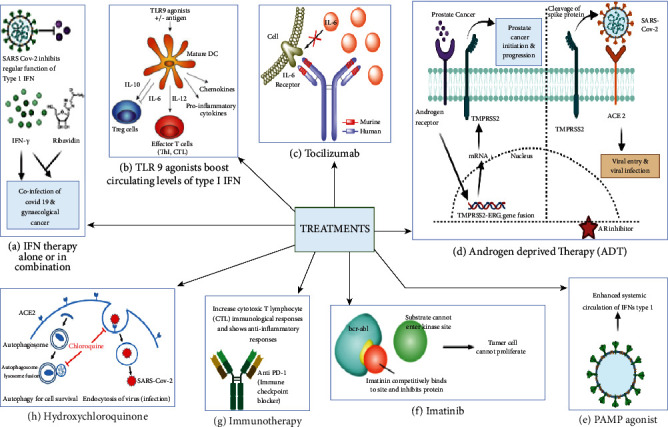
The schematic representation showing different types of available treatments to fight against the coinfection of COVID-19 and gynaecological cancer. (a) Interferon-gamma alone or in combination with other drugs such as ribavidin, remdesivir, lopinavir, or ritonavir, and hydroxychloroquine can be a promising strategy to treat the coinfection. (b) TLR 9 agonists can be used to boost the circulating level of type 1 INF. (c) Tocilizumab is an antihuman IL-6R monoclonal antibody and can be used to reduce the pulmonary complications raised by SARS-CoV-2. (d) Androgen-deprived therapy has been used in the treatment of prostate cancer as well as COVID-19 infection in males. (e) Pathogen-associated molecular pattern (PAMP) agonists can enhance the systemic circulation of IFN type 1. (f) An anticancer drug, imatinib can be used to reduce the effect of COVID-19 as well as inhibit tumour cell proliferation. (g) Immunotherapy is a promising treatment for both COVID-19 infection and cancer. (h) Hydroxychloroquine is another drug that showed promising results in the treatment of coinfection.

Certain proinflammatory cytokines such as pro-IL1 *β* and pro-IL-18 are activated by caspase 1 for triggering an inflammatory response via pyroptosis in an IL-1 and IL-18-dependent manner [60], as explained in Figures 5 and 6. The host experiences symptoms like fever and headache as a result of massive macrophage and cytokine infiltration. The functional ability of the lungs along with its alveolar capacity is diminished as bronchoalveolar lavage fluid accumulates causing breathing difficulties, hypoxaemia, and pneumonia as symptoms. The systemic dissemination of the virus causes several secondary infections including CNS, digestive and cardiovascular systems along with the renal system, and ultimately causes multiple organ failures via ARDS [61]. The sequence of events that occur in COVID-19 aids in the understanding of the molecular mechanism of COVID-19.

The genome of coronavirus constitutes a molecular weight of 26–32 kb belonging to the b-CoV strain family [62]. It possess ten ORFs which are primarily constituted of structural proteins, namely, spike protein (S), nucleocapsid (N), and envelope (E) as well as membrane proteins (M) and are henceforth accompanied with the viral RNA which is being translated into big polyproteins, that make up two-thirds of the ORFs. These polyproteins are relocated in the RER, wherein the viral transcription and replication take place [62]. The S proteins are in charge of the binding of the virus and its entrance inside the host. There are three mechanisms that occur upon the entry of virus which include recognition by ACE-2 and TMPRSS2 receptors, cytokine storm, and hyperactivation of coagulopathy as explained in Figure 5. Once within the host body, the virus binds to the TMPRSS2 and ACE-2 receptors individually, making it easier to detect. While TMPRSS2-binding-activated glycoproteins promote viral penetration into host cells, ACE-2 binding promotes virus uptake by host cells leading to cytokine storm in patients afflicted with COVID-19 and gynaecological cancer [62, 63]. This in turn causes hyperactive coagulopathy in fibrinogen, antithrombin III, and D-Dimers-dependent manner depicting the molecular link in-between COVID-19 and gynaecological cancer [64].

COVID-19 infection causes coagulopathy that is predominantly prothrombotic. Coagulation factors and platelets are directly involved, as elevated cytokines affect their function. In patients with ARDS, intravascular coagulation is mainly prevalent in dying patients (71.4%) than that of the surviving patient (0.6%) [63–65]. Based upon the stage of gynaecological cancer, timely detection, and anti-cancer medication, the risk of coagulation is linked to thrombosis. The risk of thrombosis is mostly influenced by the clinical manifestations with specific symptoms like thrombotic microangiopathy and disseminated intravascular coagulation that would activate certain tumour factors including tissue factor (TF), podoplanin, plasminogen activator factor (PAI-1), cytokines, NET, and mucins [66]. The severity of coagulation is dependent on which type of gynaecological cancer. Ovarian cancer has an increased risk of coagulation, whereas breast cancer has a lower risk [63]. Identifying the risk associated with coagulation in patients with co-infection of COVID-19 and gynaecological cancer will aid in the management and treatment of patients.

COVID-19 is dependent upon the inflammatory response, oxidative stress, and pathophysiological abnormalities, all of which can have a significant impact on gynaecological cancer diagnosis and therapy options [67, [68]. In severe cases of COVID-19, these gynaecological cancer indicators were enhanced, thus depicting itself as a prognostic biomarker for detection and diagnosis of coinfection caused by COVID-19 and gynaecological cancer [69].

## 5. Gynaecological Cancer with ACE-2 Receptor in COVID-19

The virus recognizes the ACE-2 receptor by virtue of its spike protein upon entering the cell as the receptors are extensively expressed in the nasopharyngeal, gastrointestinal, respiratory, and cardiovascular tissues [70], as well as on certain haematopoietic cells such as monocytes and macrophages [71, 72]. Due to the SARS-CoV-2 infection, ACE-2 receptors activity may be reduced as viruses bind to the receptors, which in turn results in rendering them less available to carry out other functions causing promotion of the tumour phenotype in gynaecological cancer patients with a coinfection of COVID-19 [53]. It also causes the upregulation of the Zinc Finger E-Box Binding Homeobox 1 (ZEB1) which in turn induces epithelial-mesenchymal transition in the co-infected cells as it can negatively regulate ACE-2 expression. Therefore, it can be predicted that SARS-CoV-2 infection might play a role in causing a transition in the plasticity between mesenchymal phenotypes. Those cells that are coinfected with COVID-19 and gynaecological cancer depict a decreased glutamine synthesis; which is a pivotal component of epithelial-mesenchymal plasticity [73]. This correlation was validated with the expression of ACE-2 and tumour infiltration in uterine corpus endometrial carcinoma. It was demonstrated via TGCA data that ACE-2 is overexpressed in some cancers including cervical cancer [74, 75]. On the other hand, the expression of ACE-2 was observed to be strikingly reduced in the case of breast cancer [76]. This depicts the immunomodulation due to the ACE-2 in gynaecological cancer in COVID-19.

A key study had been carried out, in which Palluzi et al. followed up on 965 patients admitted between February and April 2019, for the period of three months versus 930 patients admitted in the same months for the same duration in 2020 [77]. The maximum number of the patients had been diagnosed with ovarian cancer, i.e., 67.7%, followed by 15.2% of the patients who had been diagnosed with endometrial cancer, 10.4% of the patients had been diagnosed with cervical cancer and 6.7% of the patients with other gynaecological malignancies in the year 2019 [78–80]. In 2020, 70.8% of the patients had been diagnosed with ovarian cancer, 12.6% with endometrial cancer, 10.3% with cervical cancer, and 6.3% had been diagnosed with other gynaecological cancer [81]. In terms of the number of patients by kind of malignancy, there was no statistically significant difference between the two groups (Table 2). More, clinical studies need to be carried out to correlate the detailed mechanistic relationship between COVID-19 and gynaecological cancer.

## 6. Management of Coinfection of COVID-19 and Gynaecological Cancer

Recombinant IFN*γ* and IFN*α*2b are frequently used to treat cancer either alone or in combination with other therapies whenever there is a coinfection with COVID-19 [82]. Since type I interferon response gets hampered due to SARS-CoV-2, administering IFN systemically or locally either alone or with ribavirin [50, 83, 84], remdesivir [85, 86], lopinavir or ritonavir, and hydroxychloroquine can be a promising strategy against coinfection of COVID-19 with gynaecological cancer. Utilizing PAMP agonists to enhance systemic circulation amounts of type I IFNs. Administering TLR9 agonist at subcutaneous levels can boost circulating levels of type I IFN [87].

Application of immunotherapy through utilising immune check point blockers enhances the level of cytotoxic T lymphocyte (CTL) [88] in both viral-infected and tumor cells simultaneously but unfortunately very little is known about the involvement of T cells in the severe phase of COVID-19 at this moment. T cells induces lung immunopathology implying therapeutic reactivation of the cells can aggravate the disease [89]. Contrastingly, respiratory virus-induced pneumonitis causes lowering of CTL levels which impairs the ability of T cells to eliminate inflammatory myeloid cell. This consequently results in immune check point blockers acting as anti-inflammatory agents. Inflammation is primarily dependent upon IL-6 from elevated plasma samples of the patients which correlates the co-infection of COVID-19 with that of gynaecological cancer and other malignancies [90, 91]. The National Health Commission of the People of the Republic of China has authorized tocilizumab, an anti-human IL-6R monoclonal antibody for pulmonary complications caused by SARS-CoV-2. The risk of death in patients was decreased substantially when tocilizumab was administered intravenously or subcutaneously, but this effect was not seen in cancer patients [92].

In China, patients with severe COVID-19 when administered with tocilizumab exhibited improved clinical and radiological outcomes [93] through reduced hyperactivated inflammatory immune responses [94]. It also expressed restoration of robust adaptive immunity mediated by T-cells in patients suffering from COVID-19 [95]. When COVID-19 is severe, this type of neutrophil loss might excerbate lungs inflammation. Further research highlighted concerns about the efficacy of this therapy because of the adaptibility of tumor promoting growth factor like IL-6. Thus, anti-inflmmatary drugs and interleukin inhibitors aid in the management of coinfection caused by COVID-19 and gynaecological cancer [96].

Androgen-targeted signaling contributes to the pathogenesis of breast and ovarian carcinoma significantly as they enhance the ability of cellular proliferation and angiogenesis [97, 98]. Androgen-receptor signaling increases the expression of TMPRSS2 nonprostatic tissues and can potentially contribute to increased susceptibility of male individuals to SARS-CoV-2 [99]. Androgen-deprivation therapy (ADT) accompanied with viral inhibitorsreduces the expresion of TMPRSS2 and could be utilised to treat COVID-19 patients particularly male. Therefore, ADT when used in combination with either AR inhibitors or luteinizing hormone-releasing hormone modulators could potentially act as a propylactic therapy used as an interceptive medicine against COVID-19 patients who are suffering from a high risk of the disease [100].

The ability of hydroxychloroquine (HCQ) to suppress autophagy by acting as a lysosomotropic agent and raising intralysosomal pH had been tested on autophagy-dependent cancer [101]. HCQ can impair immunological functions by interfering with antigen processing and presentation as well as cytokine generation. HCQ can prevent SARS-CoV-2 from replicating [102, 103]. Thus, HCQ can shorten the period of COVID-19 infection and its severity serves as a potential therapuetic compound that needs to be explored and investigated with upside potential for the managment of coinfection between COVID-19 and gynaecological cancer [104, 105].

**Figure 7:**
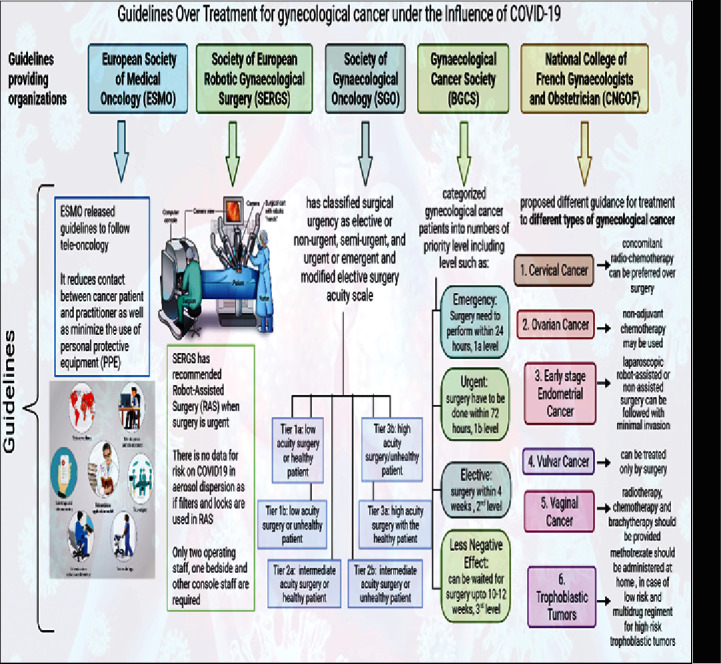
The detailed guidelines provided by the European Society of Medical Oncology (ESMO), Society of European Robotic Gynaecological Surgery (SERGS), Society of Gynaecological Oncology (SGO), Gynaecological Cancer Society (BGCS), and National College of French Gynaecologists and Obstetrician (CNGOF) on the treatment and surgery of gynaecological malignancy under the influence of COVID-19.

**Figure 8:**
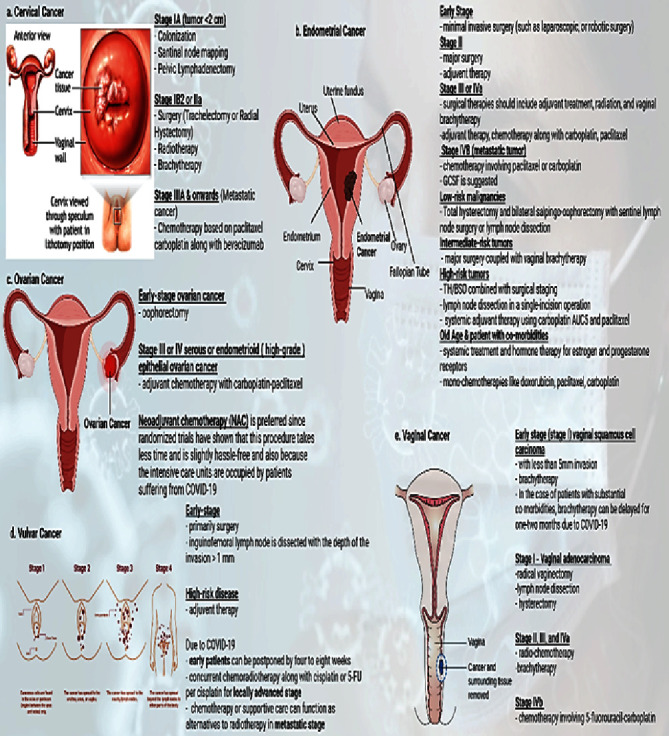
The illustration showing the impact of COVID-19 on the diagnosis and treatment of different types of gynaecological cancers.

Small compounds with anticancer and anti-SARS-CoV-2 characteristics could be explored to solve the issue of coinfection between COVID-19 and gynaecological cancer . The anticancer drug imatinib mesylate, an Abelson (ABL) kinase inhibitor, can function *in vitro* to inhibit the replication of SARS-CoV-2 [106–108]. Moreover, imatinib mesylate can activate anticancer responses mediated by T and NK lymphocytes thus indicating its immunostimulatory properties [109]. It needs to be seen whether imatinib can activate immune responses to SARS-CoV-2. Thus, these aforementioned treatments could be utilized for the management of coinfection of COVID-19 and gynaecological cancer and are illustrated in Figure 6.  Figure 7 describes the guidelines issues by medical oncology departments that need to be followed concerning gynaecological patients in the COVID-19 scenario.  Figure 8 highlights the impact of diagnosis and treatment in the COVID-19 pandemic.

## 7. Future Perspectives

The coinfection of COVID-19 along with gynaecological cancer is a potentially developing global threat to human health. As previously discussed in this review, there are no approved treatments for COVID-19. As a result, more studies would aid in the development of medicinal compounds to combat the virus. It is still unclear why certain people react differently to this coinfection. In case of patients with existing co-morbidities, such as gynaecological cancer, are more susceptible to severe COVID-19 outcomes than the general population. So, better management plans are in place to counteract the COVID-19 pandemic's negative impacts on gynaecological cancer patients. Chemotherapy or surgery should be postponed, and intense combinational therapy and a separate thero-diagnostic treatment strategy for the co-infection should be proposed. Since COVID-19 is an emerging virus in people around the world alike MERS-COV, many more cross-species infections are likely in the future, regardless of how advanced medical professions are. It is also critical to keep an eye out for additional viruses to improve our preparedness for future epidemics. To provide successful treatment, it is critical to better understand the role of immunological dysregulation in COVID-19 and gynaecological cancer. Clinical trials are being conducted on several medications, either alone or in combination. No drugs yet have been clinically useful and thus the aforementioned drugs may be considered standard of care yet. While waiting for therapeutic advances, every effort should be done to safeguard cancer patients against SARS-CoV-2 and to ensure that gynecologically cancer care is not disrupted. Moreover, herein, we have elucidated a novel interlink of the molecular cascade that would provide us and researchers with a wide spectrum to design potential target sites for inhibitors and drug targets.

## 8. Conclusions

Thus, this review reveals a bidirectional molecular relationship between COVID-19 infection and gynaecological cancer while examining the pathobiology of COVID-19 in gynaecological cancer, the risks associated with comorbidity, the overall impact on host immunity and treatment recommendations for COVID-19-infected cancer patients. This review primarily discusses the biological connection between COVID-19 and gynaecological cancer, as well as its association with the ACE-2 receptor, and how gynaecological cancer impacts COVID-19 during surgery, diagnosis, and treatment. Moreover, it presents with certain therapeutic options that could be utilized to overcome the co-infection of COVID-19 and gynaecological cancer. Furthermore, this review aids in laying the foundation for the guidelines issued by medical oncology departments that need to followed concerning gynaecological patients with COVID-19.

## Figures and Tables

**Table 1:** Clinical features distinguishing noncancer patients with COVID-19 against gynaecological cancer patients with coinfection of COVID-19.

Clinical features	Similarity or difference	Noncancer patient with COVID-19	Gynaecological cancer patient with COVID-1919	References
Clinical characteristics	Similar	Fever (88.7%)	Fever (78% to 83.6%)	[24, 43–46]
		Cough (67.8%)	Dry cough (74.3% to 80.7%)	
		Diarrhoea (3.8%)	Diarrhoea (12%)	
	Different	Sputum	Sputum	[47]
		Melena	Melena (78.4%)	

Radiographical findings (chest CT imaging)	Similar	Ground-glass opacity (65%)	Ground-glass opacity (69% to 75%)	[24, 44, 45, 48]
		A typical viral pneumonia (63%)	A typical viral pneumonia (65%71%)	
		Patchy consolidation (50%)	Patchy consolidation (46.3%)	
	Different	Bilateral lung involvement (51.8%)	Bilateral lung involvement (78.8%); a typical viral pneumonia	[49]

**Table 2:** Case reports of COVID-19 cases with gynaecological cancer.

Case distribution		February–April 2020 (%)	Reference
No. of patients	965	930	

Types of malignancy			
Ovarian cancer	653 (67.7)	658 (70.8)	[77, 78]
Endometrial cancer	147 (15.2)	117 (12.6)	[78–80]
Cervical cancer	100 (10.4)	96 (10.3)	[78, 81]
Other gynaecological malignancies	65 (6.7)	59 (6.3)	[78]

## Data Availability

No data were used to support this study.
